# Nonassociative Learning Promotes Respiratory Entrainment to Mechanical Ventilation

**DOI:** 10.1371/journal.pone.0000865

**Published:** 2007-09-12

**Authors:** Shawna M. MacDonald, Gang Song, Chi-Sang Poon

**Affiliations:** Harvard-Massachusetts Institute of Technology Division of Health Sciences and Technology, Massachusetts Institute of Technology, Cambridge, Massachusetts, United States of America; Duke University, United States of America

## Abstract

**Background:**

Patient-ventilator synchrony is a major concern in critical care and is influenced by phasic lung-volume feedback control of the respiratory rhythm. Routine clinical application of positive end-expiratory pressure (PEEP) introduces a tonic input which, if unopposed, might disrupt respiratory-ventilator entrainment through sustained activation of the vagally-mediated Hering-Breuer reflex. We suggest that this potential adverse effect may be averted by two differentiator forms of nonassociative learning (habituation and desensitization) of the Hering-Breuer reflex via pontomedullary pathways.

**Methodology/Principal Findings:**

We tested these hypotheses in 17 urethane-anesthetized adult Sprague-Dawley rats under controlled mechanical ventilation. Without PEEP, phrenic discharge was entrained 1∶1 to the ventilator rhythm. Application of PEEP momentarily dampened the entrainment to higher ratios but this effect was gradually adapted by nonassociative learning. Bilateral electrolytic lesions of the pneumotaxic center weakened the adaptation to PEEP, whereas sustained stimulation of the pneumotaxic center weakened the entrainment independent of PEEP. In all cases, entrainment was abolished after vagotomy.

**Conclusions/Significance:**

Our results demonstrate an important functional role for pneumotaxic desensitization and extra-pontine habituation of the Hering-Breuer reflex elicited by lung inflation: acting as buffers or high-pass filters against tonic vagal volume input, these differentiator forms of nonassociative learning help to restore respiratory-ventilator entrainment in the face of PEEP. Such central sites-specific habituation and desensitization of the Hering-Breuer reflex provide a useful experimental model of nonassociative learning in mammals that is of particular significance in understanding respiratory rhythmogenesis and coupled-oscillator entrainment mechanisms, and in the clinical management of mechanical ventilation in respiratory failure.

## Introduction

Mechanically ventilated patients in critical care who are unable to entrain their respiratory activity to the ventilator rhythm and fight the ventilator instead often require sedation or even paralysis in order to avoid lung injury and improve pulmonary ventilation [1]. Patient-ventilator interaction is a complex process that is determined not only by the clinician-prescribed ventilator settings but also the patient's moment-to-moment reaction to the ventilator-delivered breath [2], [3], [4], [5]. Although synchrony may be improved with the use of various patient-triggered ventilatory assist modes [6], [7], [8], the latter are relatively complex (and costly) and not always feasible or beneficial especially in neonates [9], [10] or during prolonged mechanical ventilation [11], [12], and may be prone to autotriggering [13]. Further complicating this process is the inevitable presence of extrinsic and/or intrinsic (auto) positive end-expiratory pressure (PEEP) [14], [15], [16], which may significantly influence the spontaneous breathing pattern [17] and hence, patient-ventilator synchrony.

The rising popularity of patient-triggered assisted ventilation is premised on the general belief that patient-ventilator synchrony is difficult if not impossible with controlled (non-patient triggered) mechanical ventilation. On the contrary, many studies in anesthetized animals or awake or sleeping humans have shown that periodic lung inflation during controlled mechanical ventilation may entrain the respiratory rhythm to the ventilation frequency or some sub-harmonics close to the intrinsic respiratory frequency [18], [19], [20], [21], [22], [23]. The ratio of the respiratory frequency and ventilation frequency, termed the rotation number, is a measure of the relative strength of the entrainment (strongest is 1∶1) [23], [24], [25], [26]. In anesthetized animals such entrainment is abolished after bilateral vagotomy [24], [25] and impaired after vagal cooling [27] indicating that it is mediated primarily by pulmonary slowly adapting stretch receptors and secondarily by pulmonary rapidly adapting receptors and/or vagal C-fibers.

The effects of extrinsic and intrinsic PEEP on respiratory-ventilator entrainment during controlled mechanical ventilation are less clear. If such entrainment is indeed mediated by phasic vagal volume input as predicted by theory [24], [25], then sustained elevation of lung volume during PEEP may disrupt entrainment by activating the classic Hering-Breuer inflation reflex (which is one of the earliest demonstrated long-loop physiological feedback regulation mechanisms in mammals) [28], [29]. However, previous studies in anesthetized animals have shown that the Hering-Breuer reflex response to sustained lung inflation or vagal stimulation is not static but exhibits time-dependent nonchemically-mediated central adaptation [30], [31], [32], [33]. Recent studies have ascribed such central adaptation to distinct forms of nonassociative learning (i.e., activity-dependent up- or down-regulation of the response to a continuous or repetitive stimulus) acting in concert: namely habituation via the nucleus tractus solitarius in dorsal medulla, and a novel form of nonassociative learning called desensitization via the classic pneumotaxic center in the dorsolateral pons [33], [34], [35], [36]. Desensitization (converse of secondary sensitization) is distinguished from habituation (converse of primary sensitization) by the explicit expression of memory rebound and recovery effects in the post-stimulation response [37]. In the rat, desensitization of the vagally-induced Hering-Breuer reflex is abolished by lesioning the pneumotaxic center or systemic administration of the noncompetitive NMDA receptor antagonist MK-801 [33], [38]. Habituation and desensitization have been likened to monophasic or biphasic neural differentiators (without or with memory rebound) or high-pass filters which selectively suppress tonic inputs in favor of phasic inputs [34], [37], [38], [39].

These recent revelations raise the intriguing possibility that vital physiological functions such as reflex modulation of the mammalian respiratory rhythm during controlled mechanical ventilation could be subject to nonassociative learning, a basic behavioral paradigm which has been widely studied in invertebrate animal models such as Aplysia or C. elegans and in certain cognitive functions such as the acoustic startle response in mammals [for review, see [37]], but rarely in a routine clinical setting. However, although habituation and desensitization of the Hering-Breuer reflex have been demonstrated during electrical stimulation of vagal pulmonary afferent fibers in rats [33], they have not been verified by direct lung inflation in these animals. Here, we show that pneumotaxic desensitization and extra-pontine habituation of the Hering-Breuer reflex are manifested during lung inflation in vagi-intact animals and indeed, play an important role in promoting respiratory-ventilator entrainment in the face of tonic vagal volume input during controlled mechanical ventilation under PEEP.

## Methods

All experimental protocols were reviewed and approved by the MIT Committee on Animal Care in accordance with published guidelines.

### Animal preparation

Experiments were performed on 17 urethane-anesthetized male Sprague-Dawley rats (300–350 g). The general procedures for animal surgery and anesthesia, mechanical ventilation and PEEP, cardiorespiratory/body temperature monitoring and phrenic nerve recording, etc. are as described previously [33], [40], [41] and are not repeated here.

### Pontine stimulation and lesion

The pneumotaxic center in the rat dorsolateral pons is as defined in [40]. A tungsten electrode (shaft diameter 0.1 mm, tip 1–3 µm, impedance 8–11 MΩ) was inserted stereotaxically into the dorsolateral pons (2.4–2.6 mm lateral from midline, −0.2–+0.2 mm from lambda level, and at depth of 7.5–8.5 mm from lambda surface). The latter was explored with electrical stimulation (100 Hz, pulse duration 0.3 ms, 30–40 µA, 10–15 sec) to determine the loci that produced the strongest respiratory inhibition. In some animals, 1-min long-train electrical stimulation (40 Hz) was delivered at those loci to test the respiratory responses before making lesions. Electrolytic lesions at such loci were made bilaterally by passing an anodal D.C. current (20–30 µA, for 30 sec). Subsequent electrical stimulation at the lesioned loci did not cause any respiratory response.

### Histology

At the end of the experiment, the animal was killed with overdose of urethane (2 g/kg) and perfused with 0.05 M PBS and 4% paraformaldehyde. The brainstem was removed, post-fixed in 4% paraformaldehyde for 7 days, and cut into 80-µm sections with a vibratome. The actual loci of lesions were examined histologically. Data were discarded if the lesions were outside the dorsolateral pons.

## Results

### Adaptations of respiratory-ventilator entrainment to PEEP before and after pontine lesions

**Figure 1:**
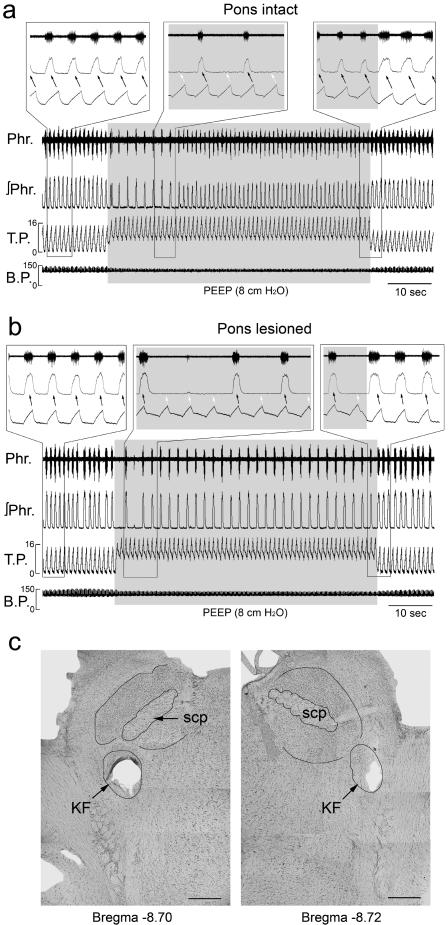
Disruption of respiratory-ventilator entrainment by positive end-expiratory pressure (PEEP) and its adaptation via nonassociative learning in a vagi-intact animal (a) before, and (b) after bilateral lesions at the dorsolateral pons. Phr, phrenic nerve discharge; ∫Phr, integrated phrenic signal; T.P., tracheal pressure (cm H_2_O) at 60 cpm; B.P., arterial blood pressure (mm Hg). Insets show expanded views of selected breaths. (c) Photomicrographs showing extent of lesions covering almost the entire Kölliker-Fuse (KF) nucleus at the left side and the ventrolateral part of this nucleus at the right side. scp, superior cerebellar peduncle. Bar: 0.5 mm.

Figure 1 illustrates the time-dependent adaptations of respiratory-ventilator entrainment to the abrupt on-off application of PEEP in a urethane-anesthetized, pancuronium-paralyzed animal with intact vagi, before and after bilateral pontine lesions. In the absence of PEEP and with pons-intact, phrenic discharge was entrained 1∶1 to the ventilator rhythm (Fig. 1a). Similar entrainment pattern was also observed before the animal was injected pancuronium. Close examination of the instantaneous tracheal pressure and phrenic discharge showed that they were phase-locked to one another, with phrenic activity beginning and ending slightly in advance of the onset and offset of inflation respectively (Fig. 1a, left and right insets).

Application of PEEP abruptly switched the entrainment to 1∶2. Phrenic activity remained phase-locked to the inflation phase every other ventilator cycle (Fig. 1a, middle inset). This 1∶2 entrainment lasted 5–30 sec before abruptly reverting to 1∶1, this time with the onset of phrenic activity lagging behind inflation although the offset still occurred before the end of inflation (Fig. 1a, right inset). Thus, the adverse effect of PEEP was “filtered” or “buffered” by respiratory adaptation although the phase relation of the entrainment was altered. PEEP also decreased the phrenic discharge duration and amplitude. Upon removal of the PEEP, the phrenic discharge immediately returned to the control level.

After pontine lesions, phrenic activity remained entrained 1∶1 to the ventilator in this animal (Figs 1b). Application of PEEP abruptly switched the entrainment to 1∶4 along with decreased phrenic discharge duration and amplitude. Thereafter, the entrainment improved to 1∶2 and remained so throughout the PEEP test. Upon removal of the PEEP, the entrainment promptly returned to corresponding pre-PEEP patterns. Also, after pontine lesions in this animal, phrenic activity showed a phase delay in its onset relative to that of inflation regardless of the presence or absence of PEEP (Fig. 1b insets). Figure 1c shows the histological definition of lesions in the pneumotaxic center.

**Figure 2:**
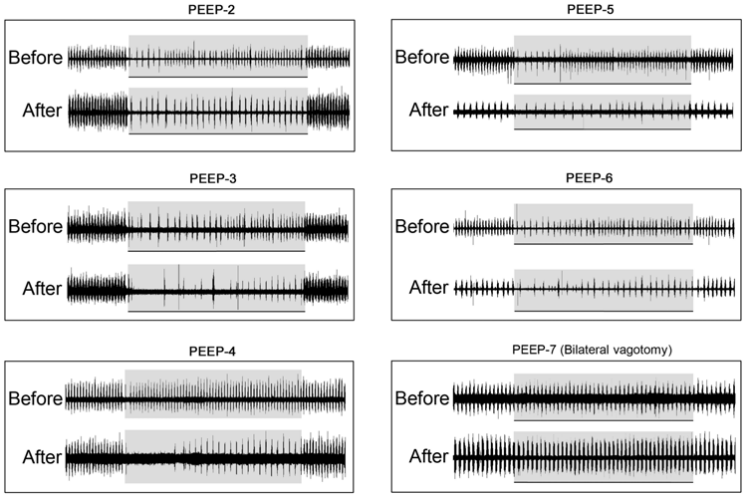
Similar tests as in Fig. 1 (PEEP-1) in five other vagi-intact animals (PEEP-2–PEEP-6) and one vagotomized animal (PEEP-7) before and after bilateral pontine lesions.

Similar effects of PEEP were observed in five other animals before and after pontine lesions (Fig. 2). In two animals (PEEP-5 and PEEP-6), respiratory-ventilator entrainment became 1∶2 after pontine lesions suggesting increased disparity between the spontaneous respiratory frequency and ventilator frequency. Upon application of PEEP the entrainment switched to 1∶4 or even higher ratios before adapting to a 1∶3 pattern. In all animals, respiratory-ventilator entrainment and its adaptation to PEEP were impaired but not abolished after pontine lesions.

In one animal under bilateral vagotomy (Fig. 2, PEEP-7), respiratory-ventilator entrainment was abolished and no discernible changes in phrenic discharge were elicited by PEEP before or after pontine lesions. Results for five other vagotomized animals were similar (not shown).

### Adaptation of respiratory-ventilator entrainment to unilateral pontine stimulation

**Figure 3:**
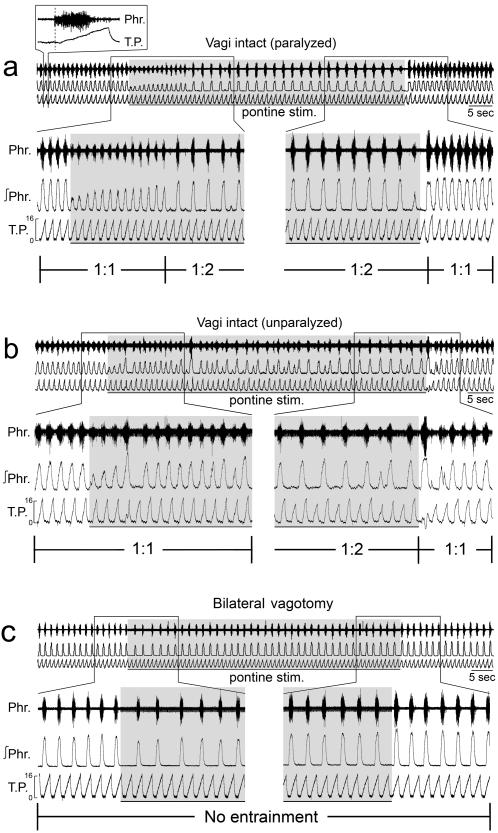
Disruption of respiratory-ventilator entrainment by unilateral stimulation at the dorsolateral pons (pontine stim.) in (a) a paralyzed, and (b) an unparalyzed animal both with intact vagi. Conventions as in Fig. 1. The response in the unparalyzed animal was more variable but the overall effects were similar to those of the paralyzed animal. (c) Absence of respiratory-ventilator entrainment before, during or after pontine stimulation in the same animal shown in (a) after vagotomy.

In five vagi-intact animals (4 paralyzed, 1 unparalyzed), unilateral electrical stimulation at the dorsolateral pons initially decreased the amplitude of phrenic discharge by>50% without affecting the respiratory entrainment (see examples in Fig. 3a, 3b). This initial inhibition lasted 15–20 sec, during which the phrenic discharge gradually adapted toward the control amplitude. Thereafter, the entrainment abruptly switched to 1∶2. Upon cessation of stimulation, the entrainment usually returned to 1∶1 immediately but occasionally could take longer.

In a paralyzed animal after vagotomy, unilateral electrical stimulation at the dorsolateral pons elicited abrupt inhibition of phrenic discharge (but to a much lesser extent than when vagi were intact) and significant prolongation of expiratory duration, both effects adapting gradually as the stimulation continued (Fig. 3c) as reported previously in vagotomized animals [42]. Entrainment was not observed before, during or after stimulation. Similar effects were seen in three other paralyzed animals after vagotomy (not shown).

## Discussion

Our results confirmed previous findings that controlled mechanical ventilation at a suitable frequency could entrain respiratory activity 1∶1 in animals with intact vagi but not after vagotomy [24], [25]. Importantly, we showed that the application of PEEP momentarily dampened the entrainment but this adverse effect was gradually buffered by respiratory adaptation via nonassociative learning. Bilateral lesions of the dorsolateral pons weakened the respiratory adaptation to PEEP whereas sustained stimulation of the dorsolateral pons weakened the entrainment independent of PEEP. These findings corroborate the notion of pneumotaxic desensitization and extra-pontine habituation of the Hering-Breuer reflex previously demonstrated during vagal stimulation [33], by showing that similar pontomedullary-mediated adaptations are also manifested during sustained lung inflation. Such central sites-specific habituation and desensitization of the Hering-Breuer reflex provide a useful experimental model of nonassociative learning in mammals that is of particular significance in understanding the mechanisms of respiratory rhythmogenesis and entrainment of coupled oscillators, and in the clinical management of mechanical ventilation in respiratory failure.

### Respiratory-ventilator entrainment is impaired by tonic vagal volume input

In a recent study in anesthetized, vagi-intact animals after acid-induced lung injury, the application of PEEP was found to promote phasic respiratory activity during neurally-adjusted ventilatory assist [43]. In those animals, the application of PEEP or vagotomy may be beneficial in restoring normal respiratory rhythm by precluding vagally-mediated lung deflation reflex secondary to the collapse of lung units following injury (see [43] and references cited therein).

In contrast, the present study showed that in animals with normal lungs respiratory entrainment to mechanical ventilation was dampened by PEEP, most probably via corresponding activation of the Hering-Breuer inflation reflex and consequent slowing of the respiratory rhythm. This is indicated by the following observations: a) the decrease of entrainment during PEEP was transient and with a similar time course as the reported central adaptation of the Hering-Breuer reflex during sustained lung inflation [30], [31]; b) the impairment worsened after bilateral lesioning of the pneumotaxic center, a site known to mediate the desensitization component of the central adaptation of the Hering-Breuer inflation reflex [33], [35]; c) electrical stimulation of the pneumotaxic region resulted in similar impairment of respiratory-ventilator entrainment as under PEEP, in agreement with previous finding that such stimulation elicits changes in the respiratory rhythm similar to the Hering-Breuer inflation reflex [42]; and d) bilateral vagotomy abolished respiratory-ventilator entrainment and corresponding influences of the pneumotaxic center and of PEEP. These observations taken together strongly support the notion that respiratory-ventilator entrainment is mediated by phasic vagal volume input and is impaired by tonic vagal volume input during PEEP due to sustained activation of the Hering-Breuer reflex.

### Habituation and desensitization of Hering-Breuer reflex during PEEP

**Figure 4:**
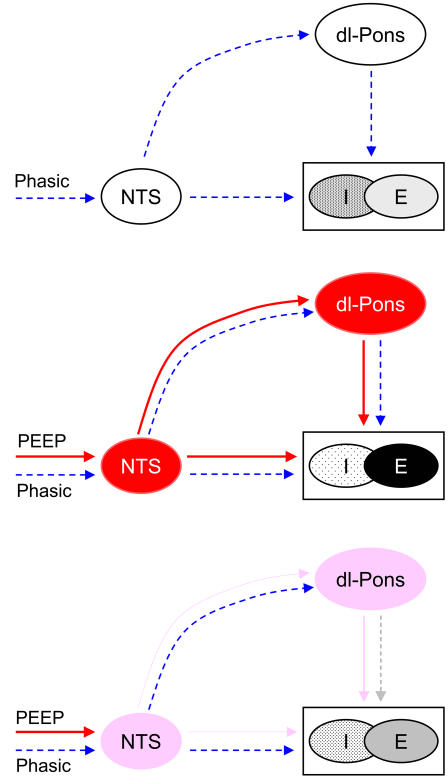
Mechanisms of respiratory-ventilator entrainment and its buffering by differentiator-type nonassociative learning. (Possible reciprocal connections for all paths are not shown.) In the absence of PEEP (top panel), phasic volume-related inputs entrain the respiratory rhythm generator (I-E) via the nucleus tractus solitarius (NTS) which also modulates the pneumotaxic center in dorsolateral pons (dl-pons). Immediately upon the application of PEEP (middle panel), tonic activities in the NTS and dl-pons elicit the Hering-Breuer reflex prolongation of expiration and shortening of inspiration, momentarily impairing entrainment. Finally, habituation of the NTS and desensitization of the pneumotaxic center (bottom panel) eventually buffer the effect of PEEP, restoring the respiratory rhythm. Sustained stimulation of dl-pons produces similar Hering-Breuer reflex and desensitization effects as PEEP.

Importantly, our results showed that this acute adverse effect of PEEP was effectively negated by nonassociative learning acting as a high-pass filter or buffer for the tonic input. This was accomplished in part through desensitization of the pneumotaxic center, but also through extra-pontine mechanisms–such as habituation of the primary pathway for vagal lung volume input [33], [35] or other vagal afferent inputs [27], or adaptation of the lung stretch receptors themselves [30]–which remained operative after pontine lesions. In this experimental setting the post-stimulation memory characteristic of pontine desensitization (which distinguishes the latter from vagal habituation [33], [37]) is not discernible because this effect contributes (if anything) to the restoration instead of disruption of respiratory-ventilator entrainment upon the removal of PEEP. Nevertheless, the specific contribution of the pons to respiratory adaptation during PEEP is clearly seen in most pontine-lesioned animals (PEEP1–PEEP4), although in some animals (PEEP5 and PEEP6) the impairment of respiratory adaptation to PEEP after pontine lesions might also involve a general weakening of respiratory-ventilator entrainment due to increased disparity between the spontaneous respiratory rhythm and the ventilator frequency. Figure 4 illustrates the suggested pontomedullary mechanisms of respiratory-ventilator entrainment and nonassociative learning adaptation that are compatible with our findings.

The important role of the pons in modulating respiratory-ventilator entrainment is also demonstrated by the dampening of entrainment during pontine stimulation. The initial inhibition and adaptation of the phrenic discharge (before the entrainment bifurcated from 1∶1 to higher ratios) during unilateral pontine stimulation in vagi-intact animals are intriguing and are in sharp contrast to the Hering-Breuer reflex-like respiratory response and adaptation to the same stimuli after vagotomy. These salient effects indicate the existence of complex adaptive processes at the right and left pneumotaxic center whereby ipsilateral and contralateral vagal and pontine inputs are integrated bilaterally in modulating the respiratory rhythm.

### Clinical relevance

Entrainment is an important physiologic phenomenon of potential clinical significance in that it allows the spontaneous (barring voluntary) respiratory rhythm to synchronize with the ventilator naturally without any external triggering device necessary. This potent effect has not been fully exploited in current assist/support-mode ventilators that rely on patient triggering, and deserves further investigation particularly in cases where the latter is not feasible or beneficial [9], [10], [11], [12]. Entrainment has been robustly demonstrated in normal humans during wakefulness or non-REM sleep or under anesthesia [19], [21], and to some extent in subjects after lung transplant [22] suggesting that other respiratory-related afferents (such as those from the chest wall) may be recruited after vagotomy to maintain entrainment. Typically, the entrained inspiratory activity precedes or lags behind machine inflation depending on whether spontaneous respiratory frequency is higher or lower than the ventilator frequency [21]. Therefore, suitable choice or servo-control of ventilator frequency to match the spontaneous respiratory frequency is crucial for optimal ventilatory unloading, as significant phase shift in the entrained respiratory activity may decrease ventilatory efficiency and increase patient discomfort.

Although the important role of phasic lung volume input in mediating respiratory-ventilator entrainment is well established, the efficacy of the Hering-Breuer reflex in humans remains controversial. It has been suggested that acute lung inflation or elevation of end-expiratory lung volume or local anesthetic blockade of the vagi has little or no immediate effect on the respiratory rhythm in awake or sleeping humans [44], [45], [46], [47], even though pulmonary stretch receptor activity remains intact [48] and the Hering-Breuer reflex has been shown to exert significant influence on the expiratory duration in humans [49], [50]. For patients with acute respiratory distress syndrome the application of PEEP is needed in order to prevent lung collapse, and hence activation of the Hering-Breuer (inflation) reflex is unlikely unless the PEEP level is excessive. At any rate, the results of the present study suggest that any tonic volume-related influence on the respiratory rhythm, if at all present, may be rendered ineffective by nonassociative learning via pneumotaxic and extra-pontine pathways. Thus, respiratory-ventilator entrainment should be robust to tonic vagal input in the face of PEEP regardless of whether the Hering-Breuer reflex is fully active or not, a desirable effect that is particularly beneficial in the clinical application of mechanical ventilation.

Although the important role of the pneumotaxic center in averting apneustic breathing has been extensively documented in vagotomized animals since the discovery of this brainstem structure over eight decades ago [51], clinical case reports of apneusis are rare and often with causes unrelated to the pneumotaxic center [52], [53]. The present results provide the first direct experimental evidence demonstrating an important role for desensitization in the pneumotaxic center and habituation in extra-pontine pathways in a routine clinical setting pertaining to the management of mechanical ventilation under PEEP, absent apneusis.

## References

[1] Tobin MJ, Alex C, Fahey PJ, Tobin MJ (2006). Fighting the ventilator.. Principles and Practice of Mechanical Ventilation. 2 ed.

[2] Dick CR, Sassoon CS (1996). Patient-ventilator interactions.. Clin Chest Med.

[3] Kondili E, Prinianakis G, Georgopoulos D (2003). Patient-ventilator interaction.. Br J Anaesth.

[4] Tobin MJ, Jubran A, Laghi F (2001). Patient-ventilator interaction.. Am J Respir Crit Care Med.

[5] Tobin MJ (2001). Advances in mechanical ventilation.. N Engl J Med.

[6] Poon CS, Lebowitz HH, Sidney DA, Li SX (1997). Negative-impedance ventilation and pressure support ventilation: a comparative study.. Respir Physiol.

[7] Sinderby C, Navalesi P, Beck J, Skrobik Y, Comtois N (1999). Neural control of mechanical ventilation in respiratory failure.. Nat Med.

[8] Sharshar T, Desmarais G, Louis B, Macadou G, Porcher R (2003). Transdiaphragmatic pressure control of airway pressure support in healthy subjects.. Am J Respir Crit Care Med.

[9] Beresford MW, Shaw NJ, Manning D (2000). Randomised controlled trial of patient triggered and conventional fast rate ventilation in neonatal respiratory distress syndrome.. Arch Dis Child Fetal Neonatal Ed.

[10] Baumer JH (2000). International randomised controlled trial of patient triggered ventilation in neonatal respiratory distress syndrome.. Arch Dis Child Fetal Neonatal Ed.

[11] Thille AW, Rodriguez P, Cabello B, Lellouche F, Brochard L (2006). Patient-ventilator asynchrony during assisted mechanical ventilation.. Intensive Care Med.

[12] Chao DC, Scheinhorn DJ, Stearn-Hassenpflug M (1997). Patient-ventilator trigger asynchrony in prolonged mechanical ventilation.. Chest.

[13] Imanaka H, Nishimura M, Takeuchi M, Kimball WR, Yahagi N (2000). Autotriggering caused by cardiogenic oscillation during flow-triggered mechanical ventilation.. Crit Care Med.

[14] Oddo M, Feihl F, Schaller MD, Perret C (2006). Management of mechanical ventilation in acute severe asthma: practical aspects.. Intensive Care Med.

[15] Pepe PE, Marini JJ (1982). Occult positive end-expiratory pressure in mechanically ventilated patients with airflow obstruction: the auto-PEEP effect.. Am Rev Respir Dis.

[16] Brochard L (2002). Intrinsic (or auto-) PEEP during controlled mechanical ventilation.. Intensive Care Med.

[17] Haberthur C, Guttmann J (2005). Short-term effects of positive end-expiratory pressure on breathing pattern: an interventional study in adult intensive care patients.. Crit Care.

[18] Petrillo GA, Glass L, Trippenbach T (1983). Phase locking of the respiratory rhythm in cats to a mechanical ventilator.. Can J Physiol Pharmacol.

[19] Graves C, Glass L, Laporta D, Meloche R, Grassino A (1986). Respiratory phase locking during mechanical ventilation in anesthetized human subjects.. Am J Physiol.

[20] Muzzin S, Baconnier P, Benchetrit G (1992). Entrainment of respiratory rhythm by periodic lung inflation: effect of airflow rate and duration.. Am J Physiol.

[21] Simon PM, Zurob AS, Wies WM, Leiter JC, Hubmayr RD (1999). Entrainment of respiration in humans by periodic lung inflations. Effect of state and CO(2).. Am J Respir Crit Care Med.

[22] Simon PM, Habel AM, Daubenspeck JA, Leiter JC (2000). Vagal feedback in the entrainment of respiration to mechanical ventilation in sleeping humans.. J Appl Physiol.

[23] Baconnier PF, Benchetrit G, Pachot P, Demongeot J (1993). Entrainment of the respiratory rhythm: a new approach.. J Theor Biol.

[24] Vibert JF, Caille D, Segundo JP (1981). Respiratory oscillator entrainment by periodic vagal afferentes: an experimental test of a model.. Biol Cybern.

[25] Petrillo GA, Glass L (1984). A theory for phase locking of respiration in cats to a mechanical ventilator.. Am J Physiol.

[26] Matsugu M, Duffin J, Poon CS (1998). Entrainment, instability, quasi-periodicity, and chaos in a compound neural oscillator.. J Comput Neurosci.

[27] Muzzin S, Trippenbach T, Baconnier P, Benchetrit G (1989). Entrainment of the respiratory rhythm by periodic lung inflation during vagal cooling.. Respir Physiol.

[28] Hering E, Porter R (1970). Self-steering of respiration through the nerves vagus (circa 1868).. Breathing: Hering-Breuer Centenary Symposium.

[29] Breuer J, Porter R (1970). Self-steering of respiration through the nerves vagus (circa 1868).. Breathing: Hering-Breuer Centenary Symposium.

[30] Stanley NN, Altose MD, Cherniack NS, Fishman AP (1975). Changes in strength of lung inflation reflex during prolonged inflation.. J Appl Physiol.

[31] Grippi MA, Pack AI, Davies RO, Fishman AP (1985). Adaptation to reflex effects of prolonged lung inflation.. J Appl Physiol.

[32] Younes M, Polacheck J (1985). Central adaptation to inspiratory-inhibiting expiratory-prolonging vagal input.. J Appl Physiol.

[33] Siniaia MS, Young DL, Poon CS (2000). Habituation and desensitization of the Hering-Breuer reflex in rat.. J Physiol 523 Pt.

[34] Poon CS, Siniaia MS (2000). Plasticity of cardiorespiratory neural processing: classification and computational functions.. Respir Physiol.

[35] Poon C-S (2004). Organization of central pathways mediating the Hering-Breuer reflex and carotid chemoreflex.. Adv Exp Med Biol.

[36] Song G, Poon C-S (2004). Functional and structural models of pontine modulation of mechanoreceptor and chemoreceptor reflexes.. Respirat Physiol Neurobiol.

[37] Poon CS, Young DL (2006). Nonassociative learning as gated neural integrator and differentiator in stimulus-response pathways.. Behav Brain Funct.

[38] Poon C-S, Young DL, Siniaia MS (2000). High-pass filtering of carotid-vagal influences on expiration in rat: role of N-methyl-D-aspartate receptors.. Neurosci Lett.

[39] Young DL, Eldridge FL, Poon CS (2003). Integration-differentiation and gating of carotid afferent traffic that shapes the respiratory pattern.. J Appl Physiol.

[40] Song G, Yu Y, Poon CS (2006). Cytoarchitecture of pneumotaxic integration of respiratory and nonrespiratory information in the rat.. J Neurosci.

[41] McGuire M, Macdonald SM, Song G, Poon CS (2007). Phrenic long-term facilitation is robust to hypercapnia and hypocapnia but not hyperventilatory hypotension under PEEP.. Respir Physiol Neurobiol.

[42] Younes M, Baker J, Remmers JE (1987). Temporal changes in effectiveness of an inspiratory inhibitory electrical pontine stimulus.. J Appl Physiol.

[43] Allo JC, Beck JC, Brander L, Brunet F, Slutsky AS (2006). Influence of neurally adjusted ventilatory assist and positive end-expiratory pressure on breathing pattern in rabbits with acute lung injury.. Crit Care Med.

[44] Widdicombe JG (1961). Respiratory reflexes in man and other mammalian species.. Clin Sci.

[45] Guz A, Noble MI, Trenchard D, Cochrane HL, Makey AR (1964). Studies on the vagus nerves in man: Their role in respiratory and circulatory control.. Clin Sci.

[46] Hamilton RD, Winning AJ, Horner RL, Guz A (1988). The effect of lung inflation on breathing in man during wakefulness and sleep.. Respir Physiol.

[47] Hamilton RD, Horner RL, Winning AJ, Guz A (1990). Effect on breathing of raising end-expiratory lung volume in sleeping laryngectomized man.. Respir Physiol.

[48] Guz A, Trenchard DW (1971). Pulmonary stretch receptor activity in man: a comparison with dog and cat.. J Physiol.

[49] Gautier H, Bonora M, Gaudy JH (1981). Breuer-Hering inflation reflex and breathing pattern in anesthetized humans and cats.. J Appl Physiol.

[50] Tryfon S, Kontakiotis T, Mavrofridis E, Patakas D (2001). Hering-Breuer reflex in normal adults and in patients with chronic obstructive pulmonary disease and interstitial fibrosis.. Respiration.

[51] Lumsden T (1923). Observations on the respiratory centres in the cat.. J Physiol London.

[52] Mador MJ, Tobin MJ (1990). Apneustic breathing. A characteristic feature of brainstem compression in achondroplasia?. Chest.

[53] Saito Y, Hashimoto T, Iwata H, Takahashi K, Fukumizu M (1999). Apneustic breathing in children with brainstem damage due to hypoxic-ischemic encephalopathy.. Dev Med Child Neurol.

